# miRNA and miRNA target genes in copy number variations occurring in individuals with intellectual disability

**DOI:** 10.1186/1471-2164-14-544

**Published:** 2013-08-10

**Authors:** Ying Qiao, Chansonette Badduke, Eloi Mercier, Suzanne ME Lewis, Paul Pavlidis, Evica Rajcan-Separovic

**Affiliations:** 1 Department of Pathology and Lab Medicine, BC Child and Family Research Institute, University of British Columbia (UBC), Vancouver, BC V5Z 4H4, Canada; 2 Department of Medical Genetics, BC Child and Family Research Institute, University of British Columbia (UBC), Vancouver, BC V6H 3N1, Canada; 3 Centre for High-throughout Biology, 177 Michael Smith Laboratories, UBC, Vancouver, BC V6T 1Z4, Canada

**Keywords:** Micro RNA (miRNA), Copy number variants (CNVs), Copy number variant regions (CNVRs), Intellectual disabilities (ID), Functional pathways

## Abstract

**Background:**

MicroRNAs (miRNAs) are a family of short, non-coding RNAs modulating expression of human protein coding genes (miRNA target genes). Their dysfunction is associated with many human diseases, including neurodevelopmental disorders. It has been recently shown that genomic copy number variations (CNVs) can cause aberrant expression of integral miRNAs and their target genes, and contribute to intellectual disability (ID).

**Results:**

To better understand the CNV-miRNA relationship in ID, we investigated the prevalence and function of miRNAs and miRNA target genes in five groups of CNVs. Three groups of CNVs were from 213 probands with ID (24 *de novo* CNVs, 46 familial and 216 common CNVs), one group of CNVs was from a cohort of 32 cognitively normal subjects (67 CNVs) and one group of CNVs represented 40 ID related syndromic regions listed in DECIPHER (30 CNVs) which served as positive controls for CNVs causing or predisposing to ID. Our results show that 1). The number of miRNAs is significantly higher in *de novo* or DECIPHER CNVs than in familial or common CNV subgroups (P < 0.01). 2). miRNAs with brain related functions are more prevalent in *de novo* CNV groups compared to common CNV groups. 3). More miRNA target genes are found in *de novo*, familial and DECIPHER CNVs than in the common CNV subgroup (P < 0.05). 4). The MAPK signaling cascade is found to be enriched among the miRNA target genes from *de novo* and DECIPHER CNV subgroups.

**Conclusions:**

Our findings reveal an increase in miRNA and miRNA target gene content in *de novo* versus common CNVs in subjects with ID. Their expression profile and participation in pathways support a possible role of miRNA copy number change in cognition and/or CNV-mediated developmental delay. Systematic analysis of expression/function of miRNAs in addition to coding genes integral to CNVs could uncover new causes of ID.

## Background

MicroRNAs (miRNAs) are an abundant class of short, non-coding, endogenous RNAs that regulate gene expression at the post-transcriptional level [1,2]. The mature miRNA is a ~20-23 nucleotides long single stranded sequence, which derives from primary transcript miRNA (pri-miRNAs) [3]. One miRNA can bind to hundreds of target genes at the 3’-UTR of mRNAs (miRNA target genes), and a single miRNA target gene can be targeted by multiple miRNAs [4,5]. Based on bioinformatic predictions, up to 90% of human genes are believed to be regulated by miRNA [5]. MiRNAs have primarily been demonstrated to mediate translational repression or target gene degradation and silencing. Recently, it has been shown that they can also upregulate gene expression by targeting gene regulatory sequences [6].

Genetic polymorphisms in miRNA or their targets can add to the complexity of miRNA regulation and function. Single nucleotide polymorphisms (SNPs) in miRNA binding sequences have indeed been shown to affect miRNA-mediated gene regulation and alter the expression of target genes [7]. A low SNP density at miRNA genes exists compared to the reference human genome, suggesting a negative selection for miRNA with SNPs [8-10]. CNVs, as a major class of genomic variations, also have an effect on miRNA, as demonstrated by under-representation of miRNA in highly polymorphic CNVs compared to the reference genome [10]. In contrast, the number of miRNA target genes in polymorphic CNVs is higher than in non-CNV regions, suggesting that genes integral to polymorphic CNVs are more likely to be regulated by miRNAs, in order to counteract their expression changes due to copy number variability of the region in which they reside [9]. Multiple cancer studies show that miRNAs integral to CNVs demonstrate gain or loss at the genomic level, and are associated with expression changes for ~10% -20% miRNAs [11,12].

In addition to cancer, miRNAs are involved in other human diseases, for example, cardiovascular diseases and neurological/neurodevelopmental disorders [13,14]. Evidence for the role of miRNA in these diseases is based on identifying mutations or differential expression of specific miRNAs and/or their global expression [15-17], and the effect of genomic copy number change on miRNA function is largely unknown. In rare instances association of CNVs with miRNA expression was studied in subject with cognitive delay. For example in Down syndrome an extra copy of chromosome 21 was associated with upregulation of several miRNAs from this chromosome, while their targets are downregulated [18]. In addition, a deletion of 1p21.3 containing a miRNA MIR137 has recently been reported by Willemsen et al. [19], and resulted in downregulation of the miRNA, and upregulation of three targets in subjects with ID and congenital abnormalities. Considering the role of MIR137 and its targets in brain function (synapse maturation, morphogenesis of young neurons, and axon growth), their dysfunction is considered causative of the patients’ phenotype [19]. Finally, microdeletion of a miRNA cluster MIR17HG on chromosome 13 is associated with Feingold syndrome which includes microcephaly, skeletal abnormalities and variable levels of ID [20]. The role of this miRNA in the human phenotype is further supported by a mouse knock-out model [20].

Although the above examples demonstrate the relevance of copy number change on miRNA function and its role in human disease, these studies are focusing on one type of CNV exclusively; for example, on common CNVs from normal populations [8-10], lump of CNVs from single disease cohorts such as cancer [11,12] or autism [21], or a few individual pathogenic CNVs associated with neurodevelopmental disorders [19,20]. Considering that ~ 70% of miRNAs are expressed in brain [22], and function in neurodevelopment, neurotransmission, synaptic plasticity, neurite outgrowth and dysregulation [18,23], we aimed to characterize and understand the total presence and functions of miRNAs in CNVs detected in idiopathic ID in comparison to neurotypical controls. Five different CNV subgroups were studied for their miRNA and miRNA target gene contents: (i) *de novo*, (ii) familial, (iii) common CNVs detected in subjects with idiopathic ID, (iv) common CNVs in cognitively normal subjects, and (v) CNVs associated with syndromic ID selected from DECIPHER (Database of Chromosomal Imbalance and Phenotype in Humans using Ensembl Resources) database (http://decipher.sanger.ac.uk/). CNVs from DECIPHER were considered as positive controls. Our study is unique in serving as the first report comparing the miRNA content and function in different CNV subgroups from ID subjects and cognitively typical controls.

## Results

### CNV detection and sub-classification

Initially, we identified 24 *de novo*, 46 familial and 216 common CNVs from 213 cases with idiopathic ID, 67 common CNVs from 32 cognitively normal subjects, and 30 CNVs collected from 40 ID-related syndromic regions in DECIPHER database representing CNVs known to cause or predispose to developmental delay. All the CNVs were identified experimentally except the CNVs from DECIPHER which were retrieved from the DECIPHER database. Overlapping CNVs from the same subgroups were merged into CNVRs (CNV Regions). The final set included 22 *de novo*, 46 familial, 30 DECIPHER CNVRs, and respectively 210 and 61 common CNVRs from cases versus controls (Table 1).The genomic coverage (Build 36 hg18) for all CNVRs and the miRNAs present in each CNVR are provided in Additional file 1: Table S1-S5.

**Table 1:** Comparison of the number of miRNAs in different CNV subgroups

**CNV category**	**No. of CNVs**	**No. of CNVRs**	**Size of total CNVRs (Mb)**	**Average size of CNVRs (Mb)**	**miRNA**			
					**Total No. (#)**	**Average miRNA/Mb**	**#/CNVR**	**Weighted median No.**
***De novo***	24	22	70.5	3.21	84	1.20	3.8	0.6^a^
**Familial**	46	46	29.5	3.34	14	0.47	0.3	0^b^
**Common from case**s	216	210	105.2	0.64	67	0.64	0.3	0^b^
**Common from control cohort**	67	61	34.1	0.56	30	0.88	0.5	0^b^
**Pathogenic from DECIPHER**	35	30	100.1	0.50	90	0.90	3.0	0.8^a^

^a,b:^Significant difference was found between data a and b (P < 0.05) (a Wilcoxon signed-rank test).

### Characteristics of miRNA in different CNV subgroups

#### Number of miRNAs in different CNV groups

**Figure 1:**
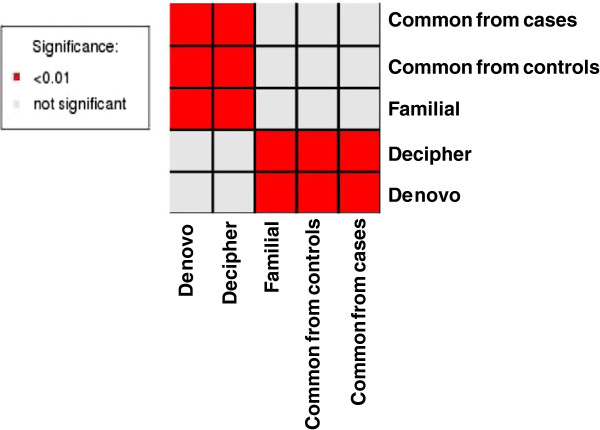
Comparison of weighted median number of miRNAs/Mb in different CNV subgroups.

The number and loci of miRNAs in different CNV subgroups were obtained using Galaxy (https://main.g2.bx.psu.edu/) and miRBase (http://www.mirbase.org/). To avoid bias caused by varying CNV size, the number of miRNA in each CNV region was weighted by the size of the CNV, and expressed as miRNA/Mb. The weighted median number of miRNAs/Mb in each CNV subgroup is shown in Table 1 and was compared among different pairs of CNV subgroups using the Wilcoxon rank-sum test. We found the median number of miRNAs/Mb is significantly higher in *de novo* and DECIPHER CNVs than familial or common CNVs from cases, and common CNVs from controls (P < 0.01) (Table 1 and Figure 1). However, the average miRNA/Mb between the common and pathogenic subgroups is comparable which is likely due to the presence of few very small common CNVs with high miRNA density (for example, 8 miRNAs within one common CNV at 16p13 (16,322,499-16,682,499).

#### Genomic coverage of miRNAs in different CNV groups

**Figure 2:**
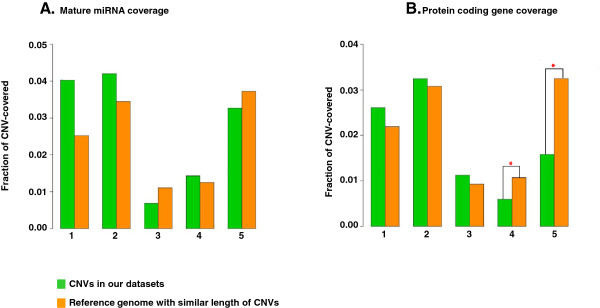
**Genomic coverage of miRNA (A) and protein coding genes (B) in different CNV subgroups.** The fraction was defined as observed number of miRNA (or protein coding genes) in each CNV group divided by the total miRNAs (or total number of protein coding genes) in human genome. The miRNA or gene fraction in CNVs was compared to the miRNA or gene fraction in the reference genome which was generated by extracting random DNA fragments with similar length to the respective CNVs 1000 times from human genome. * Indicates p < 0.05 (a Wilcoxon signed-rank test). 1: *De novo* CNVs; 2: DECIPHER CNVs; 3: Familial CNVs; 4: Common CNVs from controls; 5: Common CNVs from cases.

The genomic coverage of miRNA and protein coding genes (expressed as their number per CNV group per their total number in the whole genome) tended to be higher for *de novo* and DECIPHER CNVs, in comparison to the coverage of miRNAs and protein coding genes of randomly sampled sections from the reference genome of the same size as each of the CNV subgroups. This increase in comparison to the reference genome was the highest for miRNAs in *de novo* CNVs, but was not significant (p > 0.05, Wilcoxon signed-rank test) (Figure 2). The *de novo* and DECIPHER CNV coverage of protein coding genes was also slightly higher than expected by chance but not significant. In contrast, the protein coding genes covered a significantly smaller fraction of the common CNVs than expected by chance, i.e. when reference genome of the same size as CNVs was used (P < 0.01) (Figure 2B), in keeping with their suspected benign nature. For the familial CNVs, the observed-to-expected miRNA and protein coding gene coverage was comparable.

### Functional characteristics of miRNA from different CNV groups

#### Expression of miRNAs from CNVs

The expression profiles of miRNAs in different CNV subgroups were obtained using two web-tools, the mimiRNA database (http://mimirna.centenary.org.au/mep/formulaire.html) [24] and microRNA.org-Targets (http://www.microrna.org/microrna/home.do) [25]. Both web-tools identified a small number of miRNAs (20 ~ 30% of miRNAs in *de novo* or familial CNV groups, and ~10% of miRNAs in common CNV groups) with published expression profiles. Although the low number of miRNA with expression/function data does not allow definitive conclusions, we note that the miRNAs expressed in brain related tissues and/or having experimental evidence of involvement in nervous system dysfunction (based on manual search of PubMed) seem to be more prevalent in the *de novo*, familial and DECIPHER CNV group (~50% of all *de novo,* familial and DECIPHER CNV miRNAs with expression data) than in the common CNVs tested (0-25% of all common CNV miRNAs with expression data) (Table 2). A list of miRNAs with available expression data and brain or nervous system related function is shown in Table 3. In comparison to the common CNVs from patients, the two fold increase in the number of brain function related miRNA in familial CNV group suggests that some genes in familial CNVs could contribute to the disease process. There were no brain function-related miRNAs in common CNVs from cognitively typical controls (Table 2).

**Table 2:** Expression/Function of miRNAs in different CNV groups

**CNV category**	**No. of miRNA**	No. of miRNA with available expression data*	Brain expression/function related miRNA**
***De novo***	84	18	10 (55%)
**Familial**	14	4	2 (50%)
**Common from case cohort**	67	4	1 (25%)
**Common from control cohort**	30	3	0
**Pathogenic from DECIPHER**	90	24	11 (46%)

Notes: *indicates data collected from websites: http://www.microrna.org/microrna/ and mimiRNa (http://mimirna.centenary.org.au/mep/mir.htm).

**indicate manual PubMed search for each miRNA.

**Table 3:** MiRNAs with expression and/or function related to brain or nervous system

**#Chr**	**Start (bp)**	**End (bp)**	**miRNA ID**	**CNV feature**	Expression*	Functional relevance**	**Reference**
1	1103258	1103279	hsa-miR-200a/b	*de novo*	hsa-pancreatic islets, hsa-breast adenocarcinoma, HT29, breast malignant tumor	**olfactory neurogenesis; neuronal differentiation of neural stem/progenitor cells**	PMID:18184563; PMID: 22993445
1	1104435	1104456	hsa-miR-429	*de novo*	cancer-related	non-brain cancer related; **neuroprotective** effect in in vitro ischemia	PMID: 21684154; PMID: 20576953
5	87962684	87962705	hsa-miR-9	*de novo*	**brain, astrocytoma, neuroblastoma**	Upregulation in HD (Huntington’s disease); involved in **spinal motor neuron disease;** increased in **fmr1/fxr2 knock-out mice**	PMID: 19118166; PMID: 20616011 PMID:21957233
5	179442361	179442382	hsa-miR-340	*de novo*	**neuroblastoma**	cancer-related including **neuroblastoma**	PMID: 22797059
7	99691233	99691253	hsa-miR-25	*de novo*	**neuroblastoma**	**neural** stem cells differentiation	PMID: 21386132
7	99691438	99691460	hsa-miR-93	*de novo*	cervix-Hela	**neural** stem cells differentiation	PMID: 21386132
7	99691625	99691646	hsa-miR-106b	*de novo*	**Neurobl**-SHSY5Y_IFN, AML-THP1, kidney-embryo-HEK2	**neuronal differentiation**; pathogenesis of Alzheimer’s diseases	PMID: 21386132; PMID: 20709030
19	4770712	4770734	hsa-miR-7	*de novo*	**neuroblastoma**	Repression of alpha-synuclein accumaulation in **Parkinson’s disease**; brain cancers	PMID: 20106983; PMID: 21912681
20	61151558	61151579	hsa-miR-1	*de novo*	heart, thyroid, Ewing-sarcoma	muscle development; modulating **neurite outgrowth**	PMID: 22365735; PMID: 21170745
20	61809866	61809887	hsa-miR-124	*de novo*	**hsa-hippocamp-adult, hsa-celebellum-adult**	**neuronal differentiation and incorporation of neural-specific exons;** increased in **fmr1/fxr2 knock-out mice**	PMID: 17679093 PMID:21957233
1	94312436	94312455	hsa-miR-760	familial	hsa-**Neurobl**-SHSY5Y, hsa-Hodgkin-KMH2, hsa-**midbrain**-adult	non-brain cancer related	PMID: 22970209
15	45725296	45725317	hsa-miR-147b	familial	hsa-fibrobl-CMV, hsa-medullobl-DADY, hsa-DLBCL-DLBL3, **dendritic cel**l	rectal cancer-specific	PMID: 22850566
2	32757280	32757298	hsa-miR-558	common-case	ovary and ovary-related cancer	cancer-related including **neuroblastoma**	PMID: 21498633

Notes: *indicates data collected from websites: http://www.microrna.org/microrna/ and mimiRNa (http://mimirna.centenary.org.au/mep/mir.htm).

**indicate manual PubMed search for each miRNA.

#### Functional and pathways enrichment analysis of miRNA targets

Using the web-tool WebGestalt, we searched for pathway enrichment of miRNA target genes from different CNV subgroups compared to the reference genome. We found that approximately 8-11% of genes from *de novo* CNVs, DECIPHER CNVs or familial CNVs were targeted by miRNAs in comparison to 0-1% of genes from common CNV subgroups (P < 0.01; two-sided Fisher’s exact test) (Table 4). KEGG pathway enrichment analysis was performed using WebGestalt for identification of pathways enriched for the miRNA target genes in different CNV groups. The top 10 pathways are listed in Table 4 and contained nervous system-related pathways (Axon guidance and Neurotrophin signaling, respectively) for only *de novo* and DECIPHER CNVs (Table 4). We found that targets from both *de novo* and DECIPHER CNVs participate in MAPK signaling pathways more than expected by chance. Ubiquitin-mediated proteolysis was the only pathway found to be enriched for miRNA target genes in the familial CNV subgroup. There was no apparent pathway enrichment for the miRNA target genes from our common CNV subgroup. The miRNAs targeting the genes from the top 10 pathways for each of our CNV subgroups are also listed in Table 4.

**Table 4:** Summary of miRNA target genes within the CNV subgroups and the miRNAs targeting the CNV genes

**CNV category**	**No. of coding genes in CNVs**	**Genes in CNVs targeted by miRNA**	**Pathways enriched for miRNA target genes**	**miRNA targeting the genes in CNVs in enrichment analysis**
*De novo* from ID	737	84 (11.5%)^a^	MAPK signaling pathway	MIR-128A,MIR-128B,MIR-194,MIR-27A,MIR-27B,MIR-296,MIR-302A,MIR-302B,MIR-302C,MIR-302D,MIR-326,MIR-329,MIR-34A,MIR-34C,MIR-372,MIR-373,MIR-449,MIR-503,MIR-520A,MIR-520B,MIR-520C,MIR-520D,MIR-520E,MIR-526B,MIR-9,MIR-93
			Lysosome	
			Insulin signaling pathway	
			Axon guidance	
			Huntington’s disease	
			Hedgehog signaling pathway	
			Endocytosis	
			N-Glycan biosynthesis	
			p53 signaling pathway	
			Progesterone-mediated oocyte maturation	
Pathogenic from Decipher	838	87 (10.4%)^a^	Neurotrophin signaling pathway	MIR-196A,MIR-196B,MIR-24,MIR-320,MIR-506,MIR-493,MIR-25,MIR-32,MIR-92,MIR-363,MIR-367,MIR-512-5P,MIR-302C,MIR-30A-5P,MIR-30C,MIR-30D,MIR-30B,MIR-30E-5P,MIR-485-3P
			Renal cell carcinoma	
			MAPK signaling pathway	
			Pathways in cancer	
			Chronic myeloid leukemia	
			Chemokine signaling pathway	
			Long-term potentiation	
			ErbB signaling pathway	
			TGF-beta signaling pathway	
			Adherens junction	
Familial from ID	188	15 (8.1%)^a^	Ubiquitin mediated proteolysis	MIR-193A,MIR-193B,MIR-495,MIR-302C,MIR-198
Common from ID case cohort	454	0^b^	N/A	0
Common from control cohort	266	3 (1.1%)^b^	N/A	MIR-526B

Notes: Significant difference was found between data a and data b (P < 0.05) (a two sided Fisher’s exact test). *N/A*: not available.

## Discussion

MiRNA-mediated gene regulation is complex and genomic variations, such as CNVs and SNPs, which can modulate miRNA expression, add to this complexity. The miRNA-CNV relationship has been rarely studied and predominantly involved polymorphic CNVs found in control cohorts [9,10].

Our study is novel in its comparison of miRNA content in different classes of CNVs detected in a cohort of subjects with ID relative to cognitively typical subjects. We found a significant increase in the number of miRNAs in *de novo* and DECIPHER CNVs versus common CNVs (weighted median 0.6 and 0.8 versus 0.0, P < 0.05). In addition, the miRNAs in *de novo* CNVs were more likely to have expression in brain-related tissues or cell lines compared to the miRNAs from common CNV groups. Our collective findings suggest that miRNAs from *de novo* and putatively pathogenic CNVs could contribute to ID etiopathogenesis, in addition to coding genes integral to CNVs.

Similar to the increase in the number of miRNAs in *de novo* and DECIPHER CNVs, the number of miRNA target genes integral to these CNV types was also higher in comparison to familial or common CNVs. Lower numbers of miRNAs and miRNA target genes in common CNVs compared to the *de novo* or pathogenic CNVs suggests that they participate in processes that can tolerate functional variation. Previous studies have shown that targets for miRNAs from polymorphic CNVs tend to participate in environment-orientated processes including stimulus responses and immune responses, while the targets from non-CNV regions of the genome are enriched for fundamental biological processes such as maintenance of chromatin, chromosome segregation and nucleic acid processes [26]. In our study, genes from common CNVs that are targets for miRNAs do not show enrichment in any pathway while those from familial CNVs show enrichment in ubiquitin mediated proteolysis. In contrast, brain function related pathways such as axon guidance, Huntington’s disease, neutrophin signaling pathway, are found to be among the top 10 pathways enriched for targets from *de novo* and pathogenic CNVs. Targets from these two CNV groups also showed enrichment in the MAPK (mitogen-activated protein kinase) signaling pathway. The MAPK signaling cascade is involved in a wide variety of cellular processes and has been recently reported to be involved in ID pathogenesis [27-29]. Seven genes from *de novo* and DECIPHER CNVs which are targets for miRNAs were found to be involved in the MAPK pathway (*MAPK9, MAPT, MEF2C, MKNK2*, *PAK2*, *RPS6KA1*, and *TAOK2*). Three of them are known to be related to ID (*MAPT*[30], *MEF2C*[31,32], *PAK2*[33]). Copy number changes of these targets could affect their regulation by miRNA. Target genes for CNV miRNAs detected in subjects with autism were also found to be enriched in MAPK pathway [21].

For familial CNVs we noted that although the number of miRNAs is much lower than in *de novo* or DECIPHER pathogenic CNV groups, and not different than in common CNVs, the number of miRNA target genes was significantly higher than in common CNVs. This might suggest potential phenotypic impact of these familial CNVs. In our cohort we had two familial CNVs, predisposing to ID: 16p11.2 and 1q21.1. These CNVs are well-known for their heterogeneous phenotypes and familial or *de novo* occurrence. The 1q21.1 CNV contains a single miRNA of unknown role (miR-5087), while the 16p11.2 paternal duplication covers 2 miRNAs: miR-3680-3p and miR-3680-5p and the *de novo* 16p11.2 deletion has no miRNA content (Additional file 1: Table S2). Consequences of the variability in copy number and function of miRNA integral to these CNVs is yet unknown. Understanding the role of miRNAs and miRNA targets in familial CNVs is of interest since this type of CNV represents a significant and clinically relevant interpretational challenge that could be guided by their miRNA features.

The ultimate proof that miRNAs influence the pathogenicity of genomic changes will come from an empirical confirmation of their copy number and expression change, and influence on the expression of their targets. The limitation of our own and other studies is due to their dependence on accurate prediction of miRNA targets and the fact that miRNA numbers increase dramatically between different versions of miRBase database. Therefore, comparison between different studies in terms of miRNA content in CNVs is challenging. Furthermore, some miRNAs may still represent false positive discoveries [10]. Unfortunately, we do not have miRNA expression data from our CNVs as RNA was not available. However, a recent study by Garcia-Orti et al. [34], demonstrated 10% of 259 studied miRNAs from regions of gain and loss detected in AML had significant change in expression concordant with the type of copy number change.

A more recently published study highlighted the significance of specific CNV-miRNAs and their targets in autism [21]. It assessed the content and function of 378 autism-associated CNVs, and detected 71 miRNAs. Five miRNAS were previously reported in ASD and 3 were known to have neuronal function. In our study, among the 84 miRNAs in the *de novo* CNVs, 3 were found to be associated with neurodegenerative disorders (miRNA-7, miRNA-9, miRNA-106b) [35-37] and 1 with ID (miRNA-9) [37].

Our analysis of miRNAs and miRNA targets related to CNVs is a first attempt to evaluate their role in a patient cohort manifesting idiopathic intellectual disabilities. Additional studies of cohorts of subjects with ID would benefit further evaluation of the apparent increase in miRNA content in *de novo* CNVs demonstrated in this study. In clinical practice, the interpretation of miRNAs that occur in patient CNVs is frequently challenging, particularly if they are the only genes that are included in the CNV. With global investigations of miRNAs in subjects with ID, followed by their expression analysis, our understanding of the role of miRNA in ID pathogenesis will be further improved.

## Conclusions

Our findings support a possible role of miRNA copy number change in cognition and/or CNV-mediated developmental delay based on increased number of miRNA and miRNA target genes in *de novo* versus common CNVs in subjects with ID as well as their expression profile and participation in pathways. Systematic analysis of expression/function of miRNAs in addition to coding genes integral to CNVs could uncover new causes of ID.

## Methods

### Subjects

213 subjects with idiopathic ID were recruited for array CGH analysis by clinical geneticists across Canada. Selected individual or groups of cases were reported previously [38-42]. A cohort of 32 cognitively normal subjects had array testing as internal control samples. All of the subjects were tested by either Agilent 105 K Oligo array (227 subjects) or NimbleGen array (18 subjects). Forty syndromic genomic regions were selected from the DECIPHER database (http://decipher.sanger.ac.uk/) [43] and represented patients with neurodevelopmental delay associated with CNV findings of established potential for pathogenicity. The use of the DNA from these patients in our cohort was approved by the Committee for Ethical Review of Research involving Human Subjects, University of British Columbia. All subjects gave written informed consent for participation in the study and anonymized data were used for CNV/miRNA analysis.

### Array CGH

Genomic DNA was extracted from peripheral blood using PUREGENE DNA Isolation Kits (Gentra, Minneapolis, MN). A pool of normal male or female control DNAs (Promega, Madison, WI) was used as reference DNA matching the sex of the proband samples.

Agilent 105 K array analysis was performed according to the protocol provided by the company (version 4.0, June 2006, Agilent Technologies, CA, USA) [44]. Feature Extraction software (version 8.1.1.1, Agilent Technologies) rendered image analysis using the manufacturer’s recommended settings (CGH_v4_95) and human genome assembly hg18. The minimum absolute average of log2 ratio was 0.25.

Higher-resolution 385 K oligonucleotide genome array CGH was performed courtesy of NimbleGen. Array log2 ratio > ±0.2 was used for a segmentation (region).

For both the Agilent and Nimblegen array platforms, ≥3 consecutive probes were required for a significant CNV call. CNVs that overlapped in genomic coverage were considered to represent the same CNV loci.

### Types of CNVs

All detected CNVs were grouped into 3 subgroups (de novo, familial and common CNVs) based on the criteria described previously [45]. Briefly, CNVs completely overlapping with variants reported in at least two studies in the Database of Genomic Variants (DGV) (http://projects.tcag.ca/variation/) or in our internal controls were considered common CNVs; CNVs that overlapped partially (<50%) or did not overlap with CNVs reported in the DGV or our internal controls were called rare CNVs including de novo (not detected in proband’s parents) and familial CNVs (inherited from either of parents). All unique CNVs (*de novo* and familial) were confirmed by an independent secondary method (such as FISH method) and only single copy gains or losses were identified.

### Bioinformatics analysis

The complete list of miRNAs in the whole genome was downloaded from miRBase v19 (http://www.mirbase.org/) [46], containing >2000 mature miRNAs in human. The miRNAs in different CNV subgroups were obtained by using intersecting tool in Galaxy (https://main.g2.bx.psu.edu/) [47].

The expression profiles of miRNAs were obtained by using mimiRNA database (http://mimirna.centenary.org.au/mep/formulaire.html) [24] and microRNA.org - Targets and Expression (http://www.microrna.org/microrna/home.do) [25]. Both web-tools provide experimentally derived miRNA expression data after inputting a specific miRNA name.

The genomic locations of 19,905 human protein coding genes (PCG) were extracted from the MISO database (http://genes.mit.edu/burgelab/miso/index.html). Gene content of different CNV subgroups was obtained by considering genes within or covering CNV regions.

WebGestalt2 (WEB-based GEne SeT AnaLysis Toolkit V2) (http://bioinfo.vanderbilt.edu/webgestalt/) is a publicly available web-tool for functional enrichment analysis of gene sets using a web-based integrated data mining system [48]. Using hypergeometric test, the top 10 groups of protein coding genes from each CNV subgroup that are targets for miRNA were generated by this tool and ranked by adjP (p value adjusted by multiple test adjustment). Among these miRNA target genes, only those with adjP < 0.05, i.e. distributed in CNVs more often than expected when compared to reference genome, were selected for Kyoto Encyclopedia of Genes and Genomes (KEGG) pathway enrichment analysis. Similarly, the top ten KEGG pathways enriched for miRNA target gene were generated by the same tool for each CNV subgroup. Only the top ten enriched pathways with adjP < 0.05 were selected and compared between different CNV subgroups. The WebGestalt2 tool was also used to identify miRNAs that target the target genes for each CNV group.

### Statistical analysis

#### All statistical analyses were performed in R 2.12

*MiRNA and protein coding gene (PCG) coverage relative to random distribution:* for each CNV/CNVR subgroup we generated a set of random CNV/CNVRs with similar length distribution and total genome coverage. The number of miRNA regions and PCG regions affected by these random CNV/CNVRs was assessed. This operation was repeated 1000 times for each of the 5 CNV subgroups, generating a series of values that served as a measurement of the miRNA and PCG coverage of CNV/CNVRs under a random distribution. We performed a two-tailed test to compute the p value of the actual miRNA and PCG coverage of the 5 CNV/CNVR subsets. The p values were calculated as the number of values falling above (or below) the observed miRNA and gene coverage of the actual CNVRs datasets.

*Comparison of miRNA and PCG content:* we computed the number of miRNA and PCG in the individual CNVs relative to their size i.e. the number of miRNA and PCG per Mb. We compared the subgroups to each other using a Wilcoxon signed-rank test to assess whether a group has a significantly different miRNA and PCG density.

In order to determine the significance of the number miRNA related to brain function, we compared the fraction of brain-function related miRNA in the pool of available CNV-miRNA between each pair of CNV subgroups. The statistical p value was computed using a two-sided Fisher’s exact test. Similarly, we performed two-sided Fisher’s exact tests to compare the fraction of genes targeted by miRNA in the different CNV subgroups using the total number of genes encompassed by the CNVs.

## Abbreviations

CNVs: Copy number variations; CNVRs: Copy number variant regions; DECIPHER: Database of chromosomal imbalance and phenotype in humans using ensembl resources; ID: Intellectual disability; KEGG: Kyoto encyclopedia of genes and genomes; MAPK: Mitogen-activated protein kinase; MAPK9: Mitogen-activated protein kinase 9; MAPT: Microtubule-associated protein tau; MEF2C: Myocyte enhancer factor 2C; miRNA: MicroRNA; MKNK2: MAP kinase interacting serine/threonine kinase 2; PAK2: P21 protein (Cdc42/Rac)-activated kinase 2; PCG: Protein coding gene; pri-miRNAs: Primary transcript miRNA; RPS6KA1: Ribosomal protein S6 kinase; 90 kDa: Polypeptide 1; SNPs: Single nucleotide polymorphisms; TAOK2: TAO kinase 2; WebGestalt2: WEB-based GEne SeT AnaLysis Toolkit V2.

## Competing interests

We declare no conflict of interest in our manuscript titled as “miRNA and miRNA target genes in copy number variations occurring in individuals with intellectual disability”.

## Authors’ contributions

YQ performed data acquisition, data analysis, and drafting of manuscript; CB initiated the project and analyzed the data; EM performed statistical and bioinformatics analyses; SL recruited clinical cases and reviewed the manuscript; PP supervised statistical and bioinformatics analyses and reviewed the manuscript; ES supervised and designed the study, helped with data interpretation, and critically revised the manuscript. All authors read and approved the final manuscript.

## Supplementary Material

Additional file 1 Genomic coordinates of CNV/CNVRs and miRNAs used in this study. Click here for file
